# Modeling Driver Behavior near Intersections in Hidden Markov Model

**DOI:** 10.3390/ijerph13121265

**Published:** 2016-12-21

**Authors:** Juan Li, Qinglian He, Hang Zhou, Yunlin Guan, Wei Dai

**Affiliations:** MOE Key Laboratory for Urban Transportation Complex Systems Theory and Technology, Beijing Jiaotong University, Beijing 100044, China; 15120815@bjtu.edu.cn (Q.H.); 13251029@bjtu.edu.cn (H.Z.); 13251158@bjtu.edu.cn (Y.G.); 13251155@bjtu.edu.cn (W.D.)

**Keywords:** driver behavior, intersections, Hidden Markov Model, Baum-Welch estimation algorithm, driver assistance system

## Abstract

Intersections are one of the major locations where safety is a big concern to drivers. Inappropriate driver behaviors in response to frequent changes when approaching intersections often lead to intersection-related crashes or collisions. Thus to better understand driver behaviors at intersections, especially in the dilemma zone, a Hidden Markov Model (HMM) is utilized in this study. With the discrete data processing, the observed dynamic data of vehicles are used for the inference of the Hidden Markov Model. The Baum-Welch (B-W) estimation algorithm is applied to calculate the vehicle state transition probability matrix and the observation probability matrix. When combined with the Forward algorithm, the most likely state of the driver can be obtained. Thus the model can be used to measure the stability and risk of driver behavior. It is found that drivers’ behaviors in the dilemma zone are of lower stability and higher risk compared with those in other regions around intersections. In addition to the B-W estimation algorithm, the Viterbi Algorithm is utilized to predict the potential dangers of vehicles. The results can be applied to driving assistance systems to warn drivers to avoid possible accidents.

## 1. Introduction

Intersections are one of the major locations where safety is a big concern to drivers. For example, on United States roadways in 2010, intersection-related collisions accounted for 47% of all vehicles involved in collisions and 28% of them were fatal collisions [1,2]. The need to reduce these crashes has resulted in much research seeking to investigate and improve traffic safety when approaching intersections. Most of them focused on identifying high risk factors affecting intersection safety; traffic flow characteristics, geometric design elements, and traffic controls and operation features [3]. Regarding traffic flow characteristics, traffic volume (total volume and right-turn volume) has a significant impact on intersection safety [4,5]. Among the geometric design elements, several variables such as intersection type, size of the intersection (number of through, right-turn, and left-turn lanes), sight distances at intersections, intersection alignment, and the locations of bus stops near the intersection have been found to significantly affect the occurrence of accidents at intersections [3,4,5,6]. In addition, traffic controls and operation features (e.g., the number of phases per cycle, speed limit) also have a significant influence on intersection safety [3,4,5,7]. 

Researchers commonly use two approaches to associate intersection accidents with the aforementioned traffic-related and geometry variables: multiple linear regression and generalized linear models (GLMs) [3,5,8]. The multiple linear regression method is the most straightforward aggregate approach. However, these methods did not consider distributional properties in discrete, nonnegative, and sporadic accident data [5,7,9]. To overcome this problem, generalized linear models (GLMs), including Poisson and Negative Binomial (NB) regression models, are widely used to establish the relationship between the number of intersection accidents and the influencing factors [3,5,8,10,11,12]. Poisson regression models are sometimes considered a superior alternative to conventional linear regression models because they require a smaller number of sample data. However, these models assume the mean and variance of accident observations to be same. This assumption limits the wide use of the models because the variance of accidents always exceeds the mean in over-dispersed accident data of intersections [5,8,13,14]. NB models add an error term to overcome the accident data over-dispersion problem and offer better model performance than Poisson models [8,13,15]. However, both Poisson and NB models do not reflect inherent correlations of accident data (longitudinal intersection crash data or panel data). Therefore generalized estimating equations (GEEs), which is an extension of GLMs, have been utilized to model accident frequencies in numerous studies [16,17]. More recently, random parameter (RP) models and hierarchical models have also been utilized to model the potential heterogeneity of accident data for safety analyses [3,18,19,20,21].

A common drawback of the above studies is that they do not consider human factors. Previous research shows that nearly 95% of all traffic accidents are related to human factors (either alone or combined with other factors) [22]. In this way, researchers firstly attempt to find what human factors would affect intersection safety and lots of studies focus on the effects of personal characteristics. Among all the drivers’ personal characteristics, the most important factors are drivers’ gender and age [14]. In general, the research results have shown that male drivers are more likely to be involved in serious or fatal traffic accidents and median-aged drivers are less likely to be involved in serious or fatal traffic accidents compared with young and old drivers [23,24,25,26]. Previous studies have also shown that drivers’ attributes such as education level, income, and marital status influence traffic safety and traffic violations [27,28,29]. Subsequent studies found that social and cultural characteristics are also very crucial to traffic safety [28,30].

In fact, a major contributing factor affecting intersection safety is the drivers’ inability to correctly assess and predict the dangers that they may face. Most studies that considered the impact of the driver mostly focused on determining the relationship between the various explanatory variables mentioned above (e.g., drivers’ personal attributes, social and cultural characteristics) and the dependent variable (a serious or fatal accident) using logistic regression models. Specifically, it is the driver’s decision-making behavior that should warrant the most attention [31,32]. But only a few researchers referred to driver decision-making behaviors approaching to intersections.

Drivers’ behaviors are complex. Drivers have to perceive and interact with the status of their own vehicle and adjacent vehicles, traffic signals, road conditions, weather, and even lighting conditions [33]. Thus, drivers’ behaviors vary among the population and change over time. In doing so, drivers are adaptive to the environmental, physical, and psychological conditions. To model the stochastic driver behaviors, microscopic simulation-based models such as control models (e.g., car-following models, lane-changing models, and emergency maneuver models), cellular automation models, and intelligent driver models are widely used. Though these models can emulate vehicle movements, they consistently oversimplify intersection maneuvers and cannot represent the stochastic drivers’ decision-making process in detail. In addition, the results of these models are all simulated; thus whether they are consistent with reality is unclear.

Hidden Markov Models (HMMs) have recently been considered for use in modeling the stochastic drivers’ decision-making process because these models take drivers’ mental states into account. In HMMs, each driver’s driving process is described as a continuous trajectory which involves a set of discrete decisions made by the driver [34]. The discrete decisions which represent drivers’ decision-making behaviors are then estimated and predicted. Oliver and Pentland used contextual information and observation vehicles to recognize drivers’ mental actions [35]. Mitrovic applied an HMM to determine drivers’ intentions for numerous vehicle maneuvers using speed and acceleration [36]. Though these studies confirmed that the HMM is effective in modeling drivers’ behaviors, there is still a need to see if the HMM can be used to analyze the driving characteristics of drivers approaching intersections, especially when drivers are in the dilemma zone. The existing HMM studies have not yet provided an analysis of driver behaviors in different segments approaching intersections.

A dilemma zone is defined as a special zone before the stop line of an intersection at the onset of yellow signal. In this zone, no matter what decision (i.e., go or stop) the driver makes, he/she cannot successfully and safely pass through the intersection or stop before the stop line. It is the most hazardous portion of the intersection approach in which traffic conflicts or red-light running violations are most likely to occur [37,38]. Therefore, it’s necessary to study drivers’ behaviors in the dilemma zone.

This study aims to develop a HMM that can assess the stability and risk of driving behaviors when drivers approach intersections, especially when they are in the dilemma zone. Based on the parameters obtained from the HMM, we can determine the diversity of driving behaviors in different regions of the intersections. Additionally, we can also predict drivers’ driving actions and identify the potential risk of vehicles. The HMM, when applied with the connected vehicle and autonomous vehicle technologies, can be an effective model in providing drivers with advance warnings and helping drivers avoid possible accidents at intersections.

## 2. Methodology

### 2.1. Hidden Markov Model

The Hidden Markov Model (HMM) is a statistical Markov model in which a Markov chain with unobserved (hidden) states is used to represent the system being modeled. This model, originally developed by Leonard E. Baum and his coworkers, is an improvement to conventional Markov models [39]. In a conventional Markov model (like a Markov chain), the state of a system to be modeled can be observed and the state transition probabilities are the only parameters to be estimated when the structure of the Markov model is developed and calibrated. In a Hidden Markov Model, the state is not directly visible, but the output, derived from the state, can be observed. Each state is estimated with a probability distribution over the possible outputs. Therefore, within the Markov chain, the sequence of outputs generated by an HMM gives some information about the sequence of hidden states [40].

An HMM can be described by the following characteristics: *N*, *M*, *A*, *B* and *π*.

1. *N* is the number of possible hidden states in the model. Each individual state can be denoted as $S_{i}$, i.e., 1 ≤ *I* ≤ *N*. And the state symbol at time *t* is defined as *q_t_*.
2. *M* is the number of observable symbols per state $v_{k}$, i.e., 1 ≤ *k* ≤ *M*. And the observation symbol at time *t* is denoted as *O_t_*.
3. The state transition probability distribution $A=\left\{a_{ij}\right\}$ is denoted as:
  (1) $$a_{ij}=P\{q_{t+1}=S_{j}｜q_{t}=S_{i}\},\;\begin{array}{c} 1\leq i,j\leq N \end{array}$$
  where *a_ij_* representing the transition probability from state *S_i_* to state *S_j_*, have the following two constraints:
  (2) $$a_{ij}\geq 0,\;\begin{array}{c} 1\leq i,j\leq N \end{array}$$
  and
  (3) $$\sum_{j=1}^{N}a_{ij}=1,\;\begin{array}{c} 1\leq i\leq N \end{array}$$
  The constraint $a_{ij}\geq 0$ indicates that the state *S_i_* can reach any other state *S_j_* in one step.
4. The observation probability distribution $B=\left\{b_{j}\left(k\right)\right\}$ can be indicated as:
  (4) $$b_{j}(k)=P\{v_{k}\;\mathrm{at}\;t｜q_{t}=S_{j}\},\;\begin{array}{c} 1\leq j\leq N,1\leq k\leq M \end{array}$$
  where *b_j_(k)* represents the probability of the state value *j* at time *t* with the observation symbol *v_k_*.
5. The initial state probability distribution $\pi =(\pi_{i})$, where
  (5) $$\pi_{i}=p\{q_{1}=S_{i}\},\;\begin{array}{c} 1\leq i\leq N \end{array}$$
  *π_i_* are the probabilities of *S_i_* being the initial state in a state sequence.

An HMM model can be described by the specification of *N*, *M*, *A*, *B* and *π*. At first, the initial distribution *π* shows the initial state. Then the new state can be obtained by the state transition probability distribution *A*. Finally, the observation value is given according to the observation probability distribution *B*. 

There are several methods that can be used to estimate *N*, *M*, *A*, *B* and *π*. The primary methods are the supervised learning algorithm and the unsupervised learning algorithm. The supervised learning algorithm is an MLE method which uses observation sequence and its corresponding state sequence data. This method manually labels the training data and thus its workload is relatively large. Though the process is complicated, its results are closer to the actual situation because the results are obtained based on a large number of statistical data. The unsupervised learning algorithm, on the other hand, is a Forward–Backward algorithm that uses observation sequence. This method first randomly sets the initial value of the model parameters (λ=(A,B,π)) and then continuously updates *λ* and calculates the expectation to maximize P(O|λ).

Among all the unsupervised learning algorithms, an iterative procedure known as the Baum-Welch (B-W) algorithm is widely used [39]. It searches for an optimal solution based on expectation maximization [39,41]. The B-W algorithm estimates the model parameter λ=(A,B,π) using the forward variable $\alpha_{t}(i)$ and backward variable $\beta_{t}(i)$ to find updated values of ${\bar{a}}_{ij}$, ${\bar{\pi }}_{i}$ and ${\bar{b}}_{j}(k)$.

(6) $$\xi_{t}(i,j)=P(q_{t}=S_{i},q_{t+1}=S_{j}|O,\lambda )\begin{array}{cc} = & \begin{array}{ccc} \frac{\alpha_{t}(i)a_{ij}\beta_{t+1}(j)b_{j}(O_{t+1})}{P(O|\lambda )} & = & \frac{\alpha_{t}(i)a_{ij}\beta_{t+1}(j)b_{j}(O_{t+1})}{\sum_{i=1}^{N}\sum_{j=1}^{N}\alpha_{t}(i)a_{ij}\beta_{t+1}(j)b_{j}(O_{t+1})} \end{array} \end{array}$$

where $\xi_{t}(i,j)$ is the probability of being in state *S_i_* and *S_j_* at time *t* and *t* + 1, respectively, given the observation sequence *O* and the model parameters *λ*. $\alpha_{t}(i)$ is the partial observation sequence $O_{1},O_{2},O_{3},\cdots ,O_{t}$ given state *S_i_* at time *t*. $\beta_{t+1}(j)$ represents the remainder of the observation sequence $O_{t+1},O_{t+2},O_{t+3},\cdots ,O_{T}$ given state *S_j_* at time *t +* 1.

(7) $$\gamma_{t}(i)=P(q_{t}=S_{i}|O,\lambda )\begin{array}{cc} = & \sum_{j=1}^{N}\xi_{t}(i,j) \end{array}$$

where $\gamma_{i}(t)$ defines the probability of being in state *S_i_* at time *t* given the observation sequence *O* and the model parameters *λ*. And then, parameters can be updated as follows [34,40]:

(8) $${\bar{\pi }}_{i}=\gamma_{1}(i)$$

where $\gamma_{1}(i)$ represents the expected frequency in state *S_i_* at time *t* (*t* = 1).

(9) $${\bar{a}}_{ij}=\frac{\sum_{t=1}^{T-1}\xi_{t}(i,j)}{\sum_{t=1}^{T-1}\gamma_{t}(i)}$$

where *T* is the number of observations in the sequence. $\sum_{t=1}^{T-1}\xi_{t}(i,j)$ is the expected number of transitions from state *S_i_* to *S_j_*. $\sum_{t=1}^{T-1}\gamma_{t}(i)$ is the expected number of transitions from state *S_i_*.

(10) $${\bar{b}}_{ij}=\frac{\sum_{\begin{array}{c} t=1\\ s.t.O_{t}=v_{k} \end{array}}^{T}\gamma_{t}(j)}{\sum_{t=1}^{T}\gamma_{t}(j)}$$

where the numerator is the expected number of times in state *j* and observing symbol *v_k_*. The denominator is the expected number of times in state *j*.

Based on the above updating procedures, the new parameter $\bar{\lambda }=(\bar{A},\bar{B},\bar{\pi })$ can be obtained. The final optimal value of the parameter is acquired by iteratively using $\bar{\lambda }$ in place of *λ* and repeating updating estimation calculations.

### 2.2. Hidden Markov Driving Model

Driver behavior is affected by internal factors (e.g., drivers’ attributes, physical and psychological conditions, and perceptions and reactions to environment changes) and external factors (interferences of other vehicles, traffic controls, and weather conditions). Under such complex conditions, drivers’ decision-making processes can hardly be tracked. In this study, an HMM model is applied to describe driving behaviors of drivers when they approach intersections, which is entitled a Hidden Markov Driving Model (HMDM). This model assumes that drivers’ preferences under certain traffic conditions are fairly consistent and their resulting behaviors can be observed and derived from their previous actions. Based on the assumption, it’s possible to predict driver behaviors based on vehicles’ dynamic data and drivers’ previous performance using HMDM.

A hidden Markov chain with the HMDM is used to represent stochastic states of driver behaviors and the transition from states in one step to the states in the following step. And the probability of a state at a certain moment depends only on its accurate previous state. According to early research, driver behavior is assumed to be statistically consistent when facing certain levels of conflicts within a certain population [33]. However, drivers’ intentions cannot be observed during the driving process. Therefore, the HMM is employed to capture drivers’ intentions from several recorded sequences of the vehicle movement. The model can be used to depict drivers’ behaviors when they approach intersections. The hidden states can be estimated as the intentions of drivers (e.g., accelerate, decelerate, maintain speed, and stop), and the observation sequences can be vehicles’ dynamic data, such as the speed, headway, and acceleration. Specifically, the observed states statistically depend on the hidden states. The task of driver behavior estimation is to explore drivers’ decisions based on the observed dynamic data. The objective of the Hidden Markov Driving Model is to model the relationship between the continuous observations made by the vehicles and the discrete states representing drivers’ decisions that produced these observations.

For real-word applications, there are three basic problems to solve: evaluation, decoding, and learning problems. 

Evaluation: Given observation sequence and model λ=(A,B,π), how to calculate the probability P(O|λ).

Decoding: Given observation sequence and model, how to find the optimal hidden state sequence in a meaningful case.

Learning: Given observation sequence, how to adjust the model parameters λ=(A,B,π) to maximize the possibility P(O|λ).

The solutions to these problems are the Forward–Backward algorithm, the Viterbi algorithm, and the Baum-Welch algorithm [40,42].

Therefore, after training the basic HMM λ=(A,B,π) using the B-W algorithm, evaluation and decoding problems are solved to model stochastic driver behavior. The details are as follows:

1. Evaluation Problem

**Figure 1:**
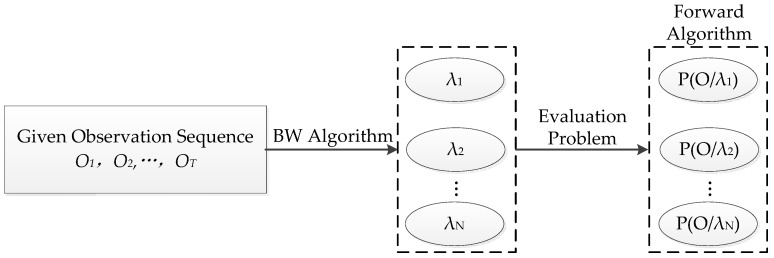
Graphica**l** illustration of the evaluation problem.

The evaluation problem refers to the situation; under the conditions that a model λ=(A,B,π) and a sequence of observation $O=(O_{1},O_{2},\cdots O_{T})$ (vehicles’ dynamic data such as the speed, headway, and acceleration) are given, how to efficiently compute the possibility that the observed sequence is produced by the given model P(O|λ), using the Forward algorithm. The illustration of this problem is shown in Figure 1.

First define the forward variable $\alpha_{t}(i)$ as:

(11) $$a_{t}(i)=P(O_{1},O_{2},\cdots ,O_{t},q_{t}=S_{i}|\lambda )$$

where $\alpha_{t}(i)$ is the partial observation sequence $O_{1},O_{2},O_{3},\cdots ,O_{t}$ given state *S_i_* at time *t*.

Step 1. Initialization:

(12) $$a_{t}(i)=\pi_{i}b_{i}(O_{1}),\;1\leq i\leq N$$

Step 2. Recursion:

(13) $$a_{t+1}(j)=[\sum_{i=1}^{N}\alpha_{t}(i)a_{ij}]b_{j}(O_{t+1}),\;1\leq i\leq T-1,1\leq j\leq N$$

Step 3. Termination:

(14) $$P(O|\lambda )=\sum_{i=1}^{N}\alpha_{T}(i)$$

Based on the calculated possibility, the stability and the risk of driver behavior approaching intersections, especially in the dilemma zone, can be determined. Specifically, the stability of driver behavior is represented by the 2-norm of the observation probability matrix *B* and the risk of driver behavior is calculated by risk index $\alpha =\sum \mathrm{lg}x_{jk}$, where *x_jk_* represents the corresponding probability of dangerous situations.

2. Decoding Problem

Based on the given model λ=(A,B,π) and a sequence of observation *O* (vehicles’ dynamic data such as the speed, headway, and acceleration), how to find the “optimal” state sequence *Q*. There are several criteria. For example, the optimal state sequence can be defined as a state sequence in which the states *q_t_* are chosen when they are individually most likely to occur. However, this could still result in an invalid state sequence [40]. 

Therefore, in this study, we adopt another criterion that is also widely used. Its main purpose is to find the single best state sequence. The single best state sequence particularly refers to maximizing the possibility P(Q|O,λ) using dynamic programming, which is also called the Viterbi algorithm.

First define the forward variable $\delta_{t}(i)$ as:

(15) $$\delta_{t}(i)=\underset{q_{1},q_{2},\cdots ,q_{t}}{\max}[P(q_{1},q_{2},\cdots ,q_{t},q_{t}=S_{i}|\lambda )]$$

where $\delta_{t}(i)$ is the highest probability along a single sequence at time *t*, which accounts for the first *t* observations and ends at state *S_i_*. And define $\varphi_{t}(i)$ to record the state sequence.

Step 1. Initialization:

(16) $$\delta_{t}(i)=\pi_{i}b_{i}(O_{1}),\;1\leq i\leq N$$

(17) $$\varphi_{t}(i)=0$$

Step 2. Recursion:

(18) $$\delta_{t}(j)=\underset{1\leq t\leq N}{\max}[\delta_{t-1}(i)a_{ij}]b_{j}(O_{t}),\;2\leq i\leq T,1\leq j\leq N$$

(19) $$\varphi_{t}(j)=\underset{1\leq t\leq N}{\arg\max}[\delta_{t-1}(i)a_{ij}],\;2\leq i\leq T,1\leq j\leq N$$

Step 3. Termination:

(20) $$P^{*}=\underset{1\leq t\leq N}{\max}[\delta_{T}(i)]$$

(21) $${q_{T}}^{*}=\underset{1\leq t\leq N}{\arg\max}[\delta_{T}(i)]$$

Step 4. State sequence backtracking:

(22) $$q_{t}^{*}=\varphi_{t+1}(q_{t+1}^{*}),\;\begin{array}{c} t=T-1,T-2,\cdots ,1 \end{array}$$

## 3. Data Description

### 3.1. Data Collection

**Figure 2:**
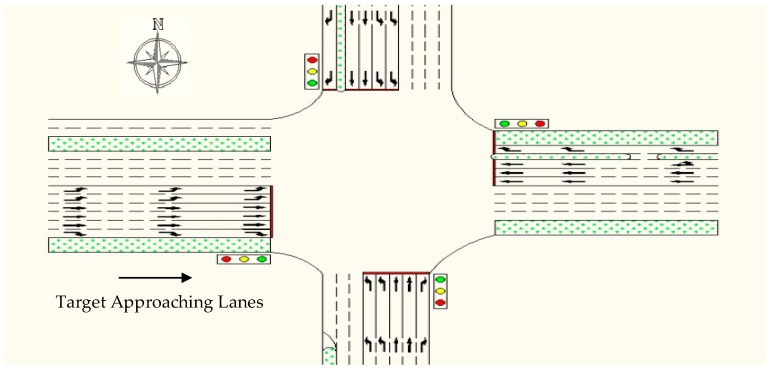
Graphical illustration of the intersection of Naoshikou Street and Xuanwumen West Street.

Data were collected in April 2015 in Beijing, China. The experiment was set at the intersection of Naoshikou Street and Xuanwumen West Street. The detailed information of the intersection is shown in Table 1 and its graphic illustration is shown in Figure 2. The though traffic volume of the straight direction is 1900 vph and the posted speed limits on all streets are 60 km/h.

**Figure 3:**
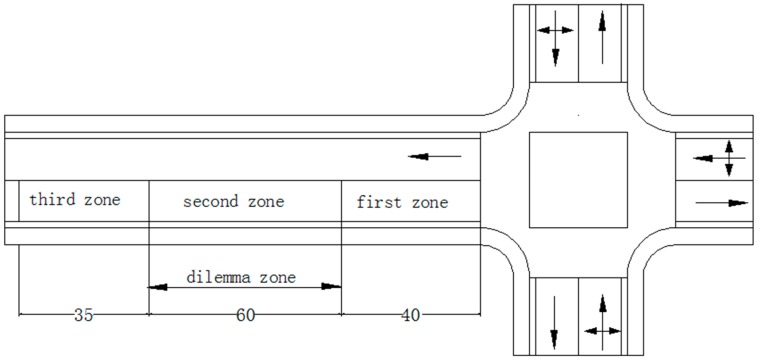
Three zones of the western approach to the intersection before the stop line.

According to previous research, the Type-II dilemma zone is defined as the area where between 10% and 90% of drivers would choose to stop at the onset of yellow signal [43]. As shown in Figure 3, the western approach to the intersection (Naoshikou Street and Xuanwumen West Street) is divided into three zones according to the definition of a Type-II dilemma zone. The two boundaries of the dilemma zone are determined by two ratios of the number of drivers with stop and go decisions to the total observed drivers at the onset of a yellow signal. The furthest boundary from the intersection indicates that 90% of observed drivers choose to stop before the stop line and 10% of observed drivers decide to go through the intersection. Based on the ratios, the approaching lanes before the stop line are divided into three zones. Specifically, the first zone is the area from the stop line to the 40 m mark line. The second zone from the 40 m mark line to the 100 m mark line is denoted as the dilemma zone and the third zone is the area between the 100 and 135 m mark lines furthest from the stop line. A total of five cameras were simultaneously employed at this intersection to record the traffic flow and the corresponding state of the traffic signal. Three of the total five cameras were set to record the three zones respectively, while the other two cameras were set to record the status of the intersection and the traffic control (one camera facing the intersection and the other camera recording the traffic signals).

### 3.2. Vehicle State Sequence

**Figure 4:**
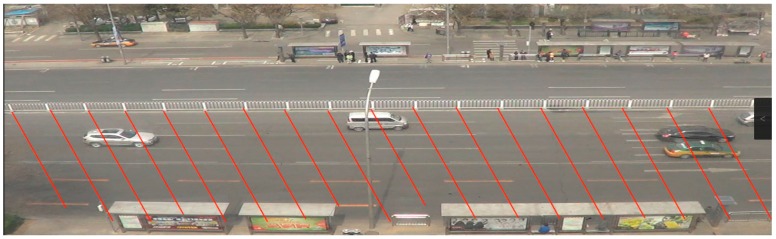
Skeleton map of detection line configuration.

Video processing software (i.e., Video Studio Pro and Adobe Premiere Pro) was used to divide the recorded images into the different regions. Vehicles’ positions were extracted by comparing images against background images with the fences as shown in Figure 4 in the center along the road in this study area. Figure 4 illustrates the skeleton map of the detection line configuration on the west approaching of the intersection (Naoshikou Street and Xuanwumen West Street). 

An image of each frame of the video can be obtained using video processing software. Thus by extracting and comparing the image of each frame, the changes in each vehicle’s location can be determined. The record of travel time of each vehicle passing each pair of the detection lines can also be ascertained; through the total number of frames. Finally, based on the distance between each pair of the detection lines (2.92 m) and the travel time, each vehicle’s speed and headway can be calculated.

Assume that a vehicle continuously passes the detection line *a*, *b*, *c*. The travel time from detection line *a* to *b* (*t_ab_*) and *b* to *c* (*t_bc_*) can be calculated:

(23) $$t_{ab}=\frac{n_{ab}}{N}$$

(24) $$t_{bc}=\frac{n_{bc}}{N}$$

where *n_ab_* and *n_bc_* are respectively the number of frames when the vehicle passes through detection line *a* to *b* and the number of frames when vehicle passes through detection line *b* to *c*. *N* is the number of frames defined to represent 1 s, which is set to 30 frames per second in this study. Then the vehicle speed at detection line *b* can be calculated:

(25) $$v_{b}=\frac{2s}{t_{ab}+t_{bc}}$$

where *s* is the distance between each pair of detection lines, which is 2.92 m. By recording the specific moment at which each vehicle exactly passes the detection line and then the lasting time between each vehicle passing two adjacent detection lines is calculated as the headway of the preceding vehicle. The queuing length and the signal light condition are also recorded.

The vehicle movement state is divided into four categories in this study; acceleration, deceleration, maintain speed, and stop. These are marked by a number from 0 to 3, respectively. This four state data can be distinguished by the vehicle’s acceleration, which can be obtained based on the changes of calculated vehicle speed.

To establish an effective observation sequence, four categories of observation data were collected; speed, headway, queue length, and signal lights. The obtained observation data were discrete to three groups respectively. Table 2 illustrates the detailed rules of discretization. The observed variables are the combination of the discrete data including 82 values, denoted by a number from 0 to 81 respectively.

Based on the data extraction process mentioned above, a total number of 256 vehicles were collected for the following analyses. After the data discrete process, a total number of 11,264 discrete data were used. All the data are ordered in a sequence and Table 3 shows part of the ordered sequence of the observation data combination.

### 3.3. Model Development

There are many factors (e.g., vehicle speed, headway of the preceding vehicle, his/her position in the traffic flow, queue length and traffic signals) that influence drivers’ decision-making behaviors. Facing such different combinations of conditions, drivers’ psychological states vary and result in different decision-making behaviors. Some drivers’ characteristics, such as the decision whether to accelerate, decelerate, maintain speed, or stop cannot be observed. Therefore, these states are defined as hidden state variables and they can be calculated by the observed variables according to the observation probability distribution *B*. Table 4 shows the hidden state variables and observed variables chosen in this study.

At the initial time, the corresponding probability values of the four states are *p*_1_, *p*_2_, *p*_3_ and *p*_4_, respectively. They are the initial probability distribution of *π*, namely, *π*_1_, *π*_2_, *π*_3_, and *π*_4_ in the HMM model. In this study, the Baum-Welch algorithm is adopted to estimate the value of *π_i_*, *a_ij_* and *b_j_(k)*. Then the supervised learning algorithm based MLE method is utilized to test the results of the B-W algorithm; that is, the estimation of the parameters of the HMM λ=(A,B,π).

(26) $${\hat{a}}_{ij}=\frac{A_{ij}}{\sum_{j=1}^{N}A_{ij}},i=1,2,\cdots ,N;j=1,2,\cdots ,N$$

(27) $${\hat{b}}_{j}(k)=\frac{B_{jk}}{\sum_{k=1}^{M}B_{jk}},j=1,2,\cdots N,k=1,2,\cdots ,M$$

(28) $${\hat{\pi }}_{i}=\pi_{i}$$

where *A_ij_* is the number of observations with the state value at time *t* and *t + 1* are *S_i_* and *S_j_* respectively. *B_jk_* is the frequency of observations with the state value *j* and the observation symbol *v_k_* at time *t*.

The whole data is divided into two parts: one for training and the other for validation. More specifically, 7480 records are randomly chosen for training, which left 3784 records for validation. 

## 4. Results and Discussion

### 4.1. Estimation of Driver Behavior

#### 4.1.1. Parameter Calibration Results

The training data are used to estimate the parameters (state transition probability matrix *A*, observation probability matrix *B*, and the initial state probability distribution probability *π*) of the three predefined zones and the whole road. 

After obtaining the parameter λ=(A,B,π) from B-W algorithm, the validation data are used to test the obtained results using Maximum Likelihood Estimation (MLE) method. Mean Absolute Percentage Error (MAPE) is calculated to measure the performance of the HMDM proposed in this study, which is the percentage of the average ratio of deviation value with actual value. And the test results show that the MAPE is about 8.17%, which shows effective performance of the HMM to model driver behavior.

#### 4.1.2. Stability of Driver Behavior

The stability of driver behavior can be described as the decisive level of the drivers’ decision-making process. It illustrates how decisive the drivers would be to make a stop/go decision in different zones of the intersection approaching lanes at the onset of a yellow signal. The more decisive driver would have a more stable driving behavior. In different zones of the intersection approaching lanes, the driver’s reaction and decision-making processes are different. Slower reaction and longer time for decision-making would lead to bad consequences such as rear-end collisions and red-light running violation. Thus it’s necessary to measure the stability of driver behavior in different zones. The 2-norm of the observation probability matrix *B* is employed to evaluate the stability of the driver behavior in different zones of the intersection approaching lanes in this study. The larger 2-norm value indicates a more decisive decision-making process, and in turn leads to a more stable driver behavior. The 2-norm of each of the three predefined zones can be calculated. Then the stability of the driver’s behavior in different zones can be compared. Shown in Table 5, the 2-norm value of the dilemma zone is smaller than that of the other two zones (i.e., the 1st zone and the 3rd zone). It turns out that the drivers in the dilemma zone may hesitate longer and act more slowly to make a stop/go decision, while drivers in the other two zones might react in a much more decisive manner.

Drivers who are in the third zone (before the dilemma zone) have stable driving behavior. For these drivers, they are far away from the stop line. Thus along the way to the intersection, they would form a complete understanding of the surrounding information, such as road conditions, traffic, and the signal indication, etc. In this way, they would better control the vehicle and have enough reaction time. Then they would be calmer at the onset of the yellow signal and have a more stable driver behavior. For drivers in the first zone (beyond the dilemma zone), they are closest to the stop line. Due to the shortest distance from the stop line, they would be more cautious of the change of the traffic signal and have fully prepared to take actions. Thus the 2-norm of drivers in this zone is the largest. However, for drivers in the dilemma zone (the 2nd zone), they have the least stable driving behavior. These drivers do not have an adequate distance to fully understand the environment to react in a timely fashion. And they don’t have an accurate forecast of the road conditions, especially the changes of the signal. They may hesitate and take longer to make certain driving decisions; thus their driving behaviors are uncertain and unpredictable.

#### 4.1.3. Risk of Driver Behavior

Dangerous behavior refers to drivers’ inappropriate actions, which may lead to potential dangers. For example, drivers still maintain their speed or even make an acceleration decision while their speed is relatively high and the headway is relatively small and there is a preceding vehicle. This non-slowdown action can defined as the dangerous behavior. Among all 82 observation combinations, six of them can be defined as the dangerous behavior: (2,2,3,1), (2,2,3,2), (2,2,3,3), (3,2,3,1), (3,2,3,2) and (3,2,3,3). Their corresponding numbers in the observed variable sequence are 43, 44, 45, 70, 71 and 72. The danger level is related to the vehicles’ positions. Therefore, the risk index $\alpha =\sum \mathrm{lg}x_{jk}$ is utilized in this study to compare the probability of dangerous behaviors of drivers in the three predefined zones of the intersection approaching lanes. In the risk index equation, *x_jk_* represents the corresponding probability of the dangerous combinations. The larger values of the risk index indicate more dangerous conditions. Table 6 shows the risk index of the whole road and the three predefined zones.

Shown in Table 6, the risk index α of the dilemma zone (the 2nd zone) is largest. It indicates that the drivers in the dilemma zone are more likely to take dangerous actions. In the dilemma zone, drivers have less time to react and may hesitate longer to make an acceleration or deceleration decision. Drivers’ slower reaction times and longer time for decision-making in the dilemma zone are potentially hazardous and may further lead to dangerous consequences. For example, though the speed is high and the headway is small, drivers still do not take action to decelerate. This decision may lead to rear-end collisions. The risk index of the third zone (before the dilemma zone) is the smallest, which indicates that these drivers are the least dangerous. They are calmer because they are farther from the stop line. With adequate distance, drivers have enough time to make a relatively correct and safe stop/go decision based on a full understanding of the traffic conditions and operation. Drivers in the first zone (beyond the dilemma zone) are closest to the stop line and most of them would first attempt to reduce their speed due to their small distance from the stop line. In this way, no matter what they choose (stop or go), the decrease in their speeds would result in a smaller likelihood of causing a dangerous consequence. Thus, the risk index of this zone is also small.

### 4.2. Predicting Driver Behavior

The dangerous state combinations are (2,2,3,1), (2,2,3,2), (2,2,3,3), (3,2,3,1), (3,2,3,2) and (3,2,3,3). Based on the observation probability matrix *B*, the most likely state value of these dangerous state combinations can be obtained. Then, through the state transition probability matrix, the most likely transition state can be selected to predict their next state value.

Taking the dangerous state combination (2,2,3,1) for example; according to the observation probability matrix of the third zone (before the dilemma zone), the probability of the hidden states (i.e., decelerating, maintain speed, and accelerating) can be calculated as 0.057, 0.029, and 0.016, respectively. Comparing these three probability values, it can be concluded that the driver is most likely to be in the deceleration state. Then, based on the state transition probability matrix, this indicates that, under the deceleration state, the state transition probabilities of the driver who decides to change the current deceleration state into the deceleration, maintain speed, acceleration, or stop state are 0.144, 0.353, 0.479, and 0.024, respectively. Compared with those four possibilities, the results show that drivers under that deceleration condition are more likely to take an acceleration decision in the next moment, since the state transition probability from deceleration to acceleration is the largest (0.479). However under the condition that the speed is high and the headway is small, it is dangerous to make an acceleration decision. Therefore when the dangerous behavior has been predicted in advance, warnings can be offered to the drivers to adjust their driving behaviors.

## 5. Conclusions

This study developed an HMDM to describe driving behaviors of drivers when the drivers are in the different segments of the intersection approaching lanes, especially in the dilemma zone. Observed vehicle dynamic data were collected to determine the structure of the HMDM. The Baum-Welch estimation algorithm was utilized to obtain the state transition probability matrix and the observation probability matrix. Effective model performance indicates that the HMDM can provide a better understanding of driver behaviors near intersections, especially in the dilemma zone at the onset of a yellow signal.

The 2-norm of the observation probability matrix and the risk index was used to evaluate the stability and risk of driver behaviors in the different segments of the intersection approaching lanes, especially in the dilemma zone. The results indicate that uncertain driver behaviors in the dilemma zone make the drivers less stable and more risky. Therefore assistance should be provided to drivers who are in the dilemma zone to avoid dangers.

Based on the results of the B-W estimation algorithm and the Viterbi Algorithm, the most likely intention of the driver can be predicted. This prediction is based on the driver’s immediate previous state (speed, headway, signal light) and the actual traffic environment; therefore it can be used in driving assistance systems to provide early warnings to the drivers who may be in danger.

This study provides a novel understanding of complex driver behaviors near intersections, especially in the dilemma zone. It can help researchers better understand driver behaviors in the intersection approaching lanes, especially in the dilemma zone, at the onset of yellow signal. In addition, predictions based on the immediately current driving state can identify potential risk and, in turn, early warnings can be provided to the target risky drivers to avoid possible hazard. It can also be applied to driver assistance systems to reduce the possibility of accidents.

## Figures and Tables

**Table 1:** Summary of intersection characteristics.

Lanes	Traffic Volume (vph)	Speed Limit (km/h)	Cycle Length (s)	Green Time (s)
straight direction	1900	60	190	35

**Table 2:** Rules of data discretization.

Speed		Headway		Queue Length		Signal Light	
Before (m/s)	After	Before (s)	After	Before	After	Before	After
≤8	1	Head Car	1	Head Car	1	Green	1
(8,16)	2	(0,6)	2	No preceding car stopped	2	Red	2
≥16	3	≥6	3	Others	3	Yellow	3

**Table 3:** Rules of the observation combination sequence (partial).

Sequence Number	Speed	Headway	Queue Length	Signal Light
1	1	1	1	1
2	1	1	1	2
3	1	1	1	3
4	1	1	2	1
5	1	1	2	2
6	1	1	2	3
7	1	1	3	1
8	1	1	3	2
9	1	1	3	3

**Table 4:** Hidden state variables and observed variables chosen in this study.

Classification	Included Variables
Hidden State Variables	Acceleration
	Deceleration
	Maintain Speed
	Stop
Observed Variables	Speed
	Headway
	Queue Length
	Signal Light

**Table 5:** 2-norm of matrix *B* of different zones of the intersection approaching lanes.

Road Range	1st Zone	2nd Zone (Dilemma Zone)	3rd Zone
2-norm of Matrix *B*	0.595	0.448	0.518

**Table 6:** Risk index of different parts of road.

Road Range	1st Zone	2nd Zone (Dilemma Zone)	3rd Zone
Risk Index *α*	−5.437	−3.343	−8.881
